# Social media insights on sepsis management using advanced natural language processing techniques

**DOI:** 10.1186/s13054-025-05344-4

**Published:** 2025-03-14

**Authors:** Ravi Shankar, Amartya Mukhopadhyay

**Affiliations:** 1 Medical Affairs – Research Innovation & Enterprise, Alexandra Hospital, National University Health System, Singapore, Singapore; 2 Division of Respiratory & Critical Care Medicine, Department of Medicine, National University Health System, Singapore, Singapore

## Comment

Early recognition and prompt initiation of appropriate treatment are critical for improving sepsis outcomes [1]. However, public insight of sepsis remains suboptimal, contributing to delays in care-seeking and worse prognoses [2]. To gain insights into public perceptions of sepsis, we analyzed 4,080 sepsis-related posts on the social media platform X.com (formerly Twitter) from January 2020 to January 2024 with advanced natural language processing (NLP) techniques.

Our multi-method approach encompassed sentiment analysis, topic modeling, aspect-based sentiment analysis, engagement analysis, and inductive thematic analysis. Our data collection utilized X.com's Academic Research Application Programming Interface (API) to gather tweets containing sepsis-related keywords in English. We preprocessed the data by removing duplicates, retweets, and non-English content. For sentiment analysis, we employed the VADER (Valence Aware Dictionary and sEntiment Reasoner) sentiment analyzer [3], specifically tuned for social media content. Topic modeling was conducted using Latent Dirichlet Allocation (LDA) [4] with optimal topic numbers determined through coherence score analysis. Our aspect-based sentiment analysis combined dependency parsing with domain-specific lexicons to identify sentiment-aspect pairs [5]. Engagement analysis incorporated retweet counts, likes, and reply metrics, while thematic analysis followed Braun and Clarke's six-phase framework [6] with two independent coders achieving strong inter-rater reliability.

Sentiment analysis revealed a complex emotional landscape with predominantly neutral (46.3%) and negative (36.1%) perceptions, highlighting the interplay between factual information-sharing and emotionally charged personal narratives. Topic modeling identified six key themes, with limited sepsis awareness (24.6% of posts) and personal experiences with sepsis (21.3%) emerging as the most prevalent (Table 1A). The dominance of these themes suggests that public understanding of sepsis is often only triggered by direct encounters with the condition, either through one's own or a loved one’s illness, underscoring the urgent need for more widespread and accessible sepsis education initiatives.

**Table 1:** Analysis of Sepsis-Related Social Media Content

Topic	Representative Words (Probability)	Proportion of Tweets
Panel A: Topic Modeling Results		
1. Sepsis symptoms and early detection	signs (0.028), symptoms (0.025), early (0.021), detect (0.019), fever (0.017), chills (0.015), confusion (0.014), breathing (0.013), heart rate (0.012), pain (0.011)	19.5%
2. Personal experiences with sepsis	family (0.032), lost (0.029), survivor (0.026), story (0.023), battle (0.021), fight (0.019), share (0.017), journey (0.015), recover (0.013), support (0.011)	21.3%
3. Sepsis prevention and risk factors	prevent (0.035), risk (0.031), factors (0.027), infection (0.024), hygiene (0.021), vaccinate (0.018), handwashing (0.016), clean (0.014), wound (0.012), immune (0.010)	16.8%
4. Treatment and outcomes of sepsis	treatment (0.040), antibiotics (0.036), hospital (0.033), ICU (0.029), organ failure (0.026), shock (0.023), survive (0.020), death (0.018), recovery (0.016), long-term (0.014)	12.5%
5. Sepsis awareness and education	awareness (0.044), education (0.039), campaign (0.035), knowledge (0.031), learn (0.027), recognize (0.024), signs (0.021), symptoms (0.019), prevention (0.016), information (0.014)	24.6%
6. Healthcare systems and sepsis management	healthcare (0.038), system (0.034), management (0.030), protocol (0.027), guidelines (0.024), training (0.021), quality (0.019), improve (0.016), standardize (0.014), coordinate (0.012)	5.3%

Predictor	Univariate Model		Regression Model	
	β	p-value	β	p-value
Panel B: Linear Regression Analysis of Factors Affecting Tweet Engagement				
Sentiment Score (Positive)	0.18	< 0.001	0.20	< 0.001
Sentiment Score (Negative)	− 0.09	< 0.01	-0.11	< 0.01
Topic 1: Sepsis symptoms and early detection	0.06	0.07	–	–
Topic 2: Personal experiences with sepsis	0.11	< 0.001	0.13	< 0.001
Topic 3: Sepsis prevention and risk factors	0.08	< 0.05	0.09	< 0.01
Topic 4: Treatment and outcomes of sepsis	− 0.03	0.28	–	–
Topic 5: Sepsis awareness and education	0.22	< 0.001	0.25	< 0.001
Topic 6: Healthcare systems and sepsis management	0.01	0.65	–	–
User Influence (In-degree Centrality)	0.35	< 0.001	0.38	< 0.001
R^2^	0.33		0.32	

Aspect-based sentiment analysis (− 1 to + 1) yielded further nuance, uncovering strong negative associations with severe clinical outcomes like "shock" (sentiment score: − 0.82) and "organ failure" (score: − 0.75), while terms like "survivors" (score: 0.62) and "awareness" (score: 0.55) were linked to positive sentiment. This duality reflects the public's recognition of both the profound threat posed by sepsis and the potential for recovery with timely intervention.

**Fig. 1:**
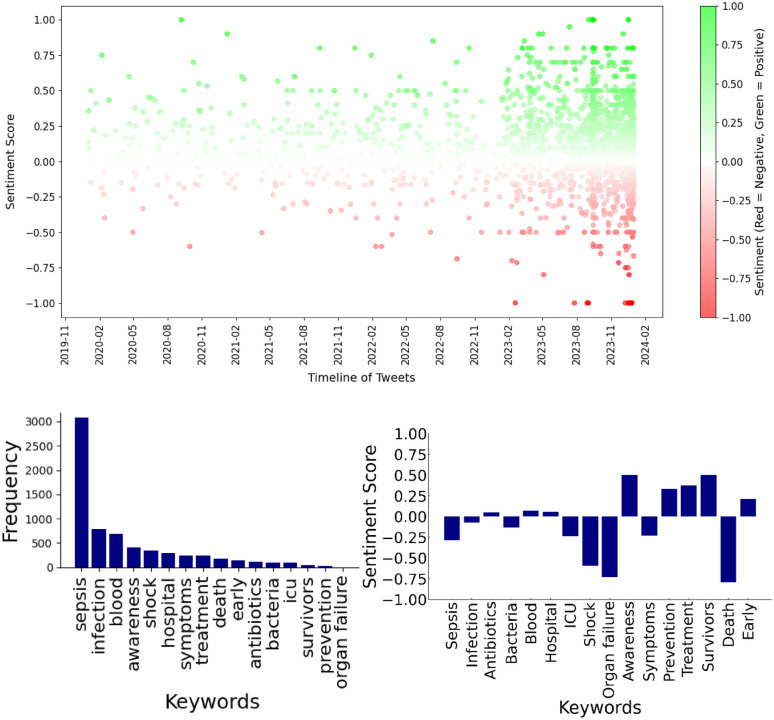
Temporal sentiment (top) and keyword analysis (bottom) of sepsis-related tweets

Figure 1 presents a comprehensive temporal and distributional analysis of sepsis-related tweets. The scatter plot (top) shows the sentiment distribution of tweets over time from 2020 to 2024, with positive sentiments indicated in green and negative sentiments in red. The temporal analysis of sentiment showed increased variability and intensity in sentiments post-2022, suggesting the impact of external events such as the COVID-19 pandemic. The bar charts (bottom) provide two complementary views: the frequency distribution of key sepsis-related terms (left) and their associated sentiment scores (right), offering insights into both how often these terms appear and their emotional impact in public discourse.

Engagement analysis provided actionable insights, revealing that tweets expressing positive sentiment, discussing sepsis awareness, and originating from influential accounts were significantly associated with higher sharing (Table 1B). These findings suggest that dissemination of educational content through key opinion leaders could enhance the reach and impact of sepsis awareness messaging.

Thematic analysis analyzed the nature of public knowledge gaps and misconceptions surrounding sepsis. A salient theme was the limited understanding of sepsis etiologies and early symptoms, with many users expressing uncertainty about the signs and associated risk factors. This lack of awareness was often only addressed through direct personal experiences of sepsis, either as a patient or a caregiver, highlighting the reactive rather than proactive nature of current sepsis education. Another prominent theme was the lack of actionable knowledge on prompt treatment-seeking behavior for suspected sepsis, with many posts reflecting a poor understanding of its time-sensitive nature and underscoring the need for early recognition and rapid care initiation. Misconceptions about sepsis prevention strategies and risk factors also emerged as a key theme, with users expressing the belief that sepsis only affects hospitalized or immunocompromised individuals ignoring many other risk factors. Furthermore, the analysis uncovered instances of stigma and misunderstanding faced by sepsis survivors, highlighting the importance of fostering greater empathy, support, and public understanding of the long-term impact of sepsis. A recurring theme was the role of social media and online resources in disseminating sepsis information and connecting affected individuals, underscoring the potential of these platforms for enhancing sepsis awareness and support. Importantly, many posts called for intensified public health efforts to promote sepsis knowledge and improve outcomes.

This study demonstrates the untapped potential of social media data as a rich source of insights into public perceptions and knowledge gaps surrounding sepsis. By harnessing advanced NLP techniques to analyze a substantial corpus of sepsis-related social media posts, we have suggested actionable strategies for crafting impactful public health messaging. Translating these insights into carefully targeted social media campaigns, in collaboration with key opinion leaders with ongoing surveillance of online discourse, represents a promising approach to enhance sepsis awareness and ultimately improving patient outcomes.

## Data Availability

Aggregated data and analysis scripts available from corresponding author. Raw data restricted by X.com Terms of Service.

## Data Availability

The datasets used and analyzed during the current study are available from the corresponding author upon reasonable request. The raw social media data cannot be shared publicly due to X.com's Terms of Service, but aggregated data and analysis scripts are available.
